# Supplementary material for: S100A8/A9 predicts response to PIM kinase and PD-1/PD-L1 inhibition in triple-negative breast cancer mouse models
Source: Commun Med (Lond). 2024 Feb 20;4:22. doi: 10.1038/s43856-024-00444-8 (PMC10879183; doi:10.1038/s43856-024-00444-8)
Supplement: Supplementary file 2 — Supplementary Information [file 43856_2024_444_MOESM2_ESM.pdf]

Supplementary Information for

S100A8/A9 predicts response to PIM kinase and PD-1/PD-L1 inhibition in triple-negative breast cancer mouse models

Lauren R. Begg,[1] Adrienne M. Orriols,[1,8] Markella Zannikou,[1] Chen Yeh,[1,2,9] Pranathi Vadlamani,[1] Deepak Kanojia,[1,10] Rosemary Bolin,[3,11] Sara F. Dunne,[4] Sanjeev Balakrishnan,[5,12] Roman Camarda,[5,13] Diane Roth,[1] Nicolette A. Zielinski-Mozny,[1,3] Christina Yau,[5] Athanassios Vassilopoulos,[1,6,14] Tzu-Hsuan Huang,[1] Kwang-Youn A. Kim,[1,2] and Dai Horiuchi[1,6,7*]

[1]Northwestern University Feinberg School of Medicine, Chicago, IL, USA
[2]Biostatistics Collaboration Center, Northwestern University, Chicago, IL, USA
[3]Center for Comparative Medicine, Northwestern University, Chicago, IL, USA
[4]High Throughput Analysis Laboratory, Northwestern University, Evanston, IL, USA
[5]University of California, San Francisco, San Francisco, CA, USA
[6]Robert H. Lurie Comprehensive Cancer Center, Northwestern University, Chicago, IL, USA
[7]Center for Human Immunobiology, Northwestern University, Chicago, IL, USA

[8]Present address: University of Florida College of Medicine, Gainesville, FL, USA
[9]Present address: Rush University Medical Center, Chicago, IL, USA
[10]Present address: Mythic Therapeutics, Waltham, MA, USA
[11]Present address: Pennington Biomedical Research Center, Baton Rouge, LA, USA
[12]Present address: Pulze.ai, San Francisco, CA, USA
[13]Present address: Novo Ventures US, Inc., San Francisco, CA
[14]Present address: AbbVie, Inc., North Chicago, IL, USA

*Corresponding author. Email: dai.horiuchi@northwestern.edu

This PDF file includes:

Supplementary Figs. 1 to 14
Supplementary Table 1

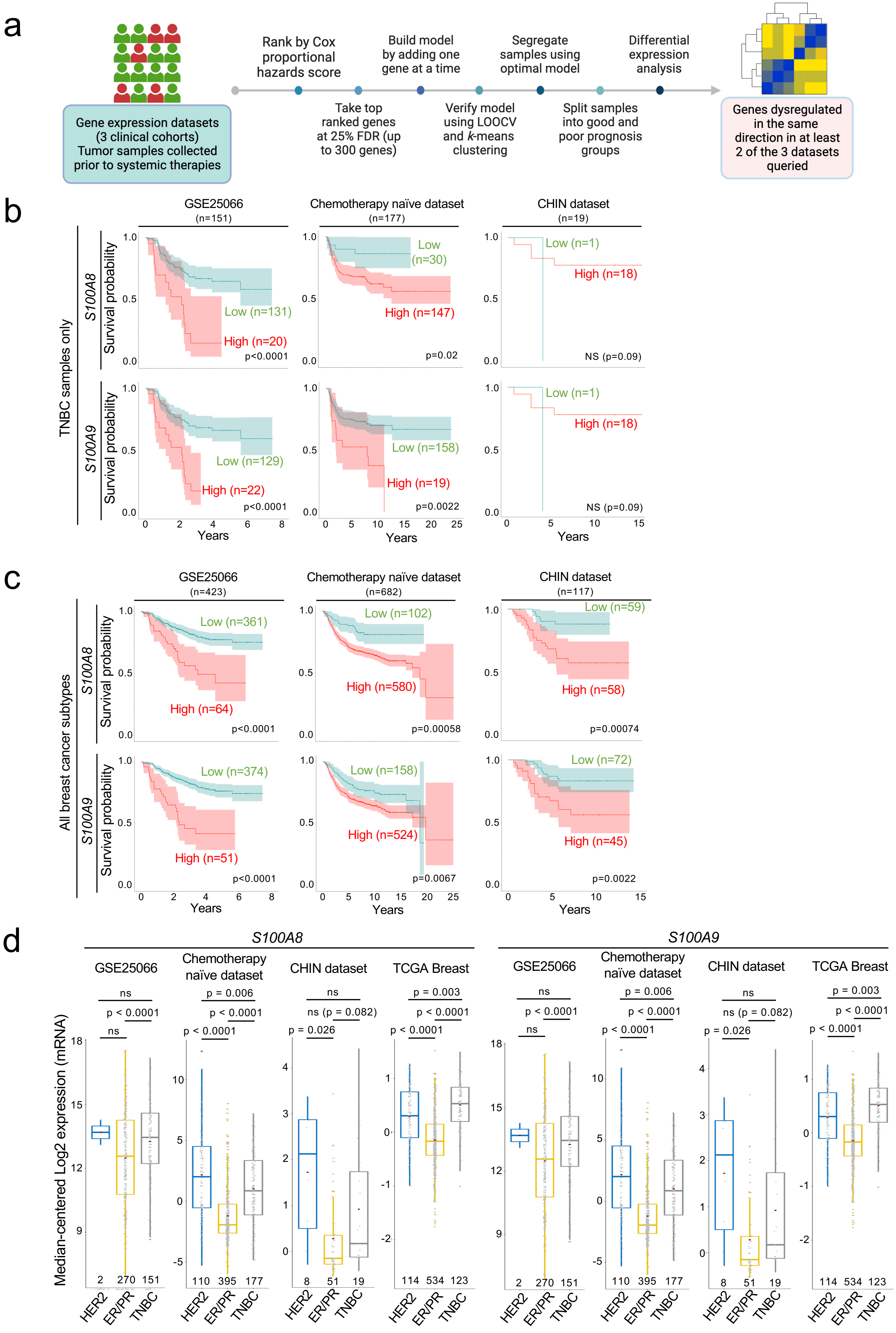

a
Gene expression datasets (3 clinical cohorts) Tumor samples collected prior to systemic therapies
Rank by Cox proportional hazards score
Take top ranked genes at 25% FDR (up to 300 genes)
Build model by adding one gene at a time
Verify model using LOOCV and k-means clustering
Segregate samples using optimal model
Split samples into good and poor prognosis groups
Differential expression analysis
Genes dysregulated in the same direction in at least 2 of the 3 datasets queried
b
TNBC samples only
GSE25066 (n=151)
Chemotherapy naïve dataset (n=177)
CHIN dataset (n=19)
S100A8
S100A9
Survival probability
Low (n=131)
High (n=20)
p<0.0001
Low (n=30)
High (n=147)
p=0.02
Low (n=1)
High (n=18)
NS (p=0.09)
Low (n=129)
High (n=22)
p<0.0001
Low (n=158)
High (n=19)
p=0.0022
Low (n=1)
High (n=18)
NS (p=0.09)
Years
c
All breast cancer subtypes
GSE25066 (n=423)
Chemotherapy naïve dataset (n=682)
CHIN dataset (n=117)
Low (n=361)
High (n=64)
p<0.0001
Low (n=102)
High (n=580)
p=0.00058
Low (n=59)
High (n=58)
p=0.00074
Low (n=374)
High (n=51)
p<0.0001
Low (n=158)
High (n=524)
p=0.0067
Low (n=72)
High (n=45)
p=0.0022
Years
d
S100A8
S100A9
GSE25066
Chemotherapy naïve dataset
CHIN dataset
TCGA Breast
Median-centered Log2 expression (mRNA)
ns
p < 0.0001
p = 0.006
p = 0.026
ns (p = 0.082)
p = 0.003
HER2
ER/PR
TNBC

Supplementary Fig. 1. Identification of *S100A8* and *-A9* as poor outcome-associated genes and their prognostic significance in TNBC and non-TNBC.

a Schematic representation of the bioinformatics methods used in this study. FDR: false discovery rate, LOOCV: leave one out cross-validation.

b, c Kaplan-Meier graphs of patients with TNBC (b) or all BC clinical subtypes (c), dichotomized by *S100A8* and *-A9* mRNA expression at an optimal threshold, from the indicated cohorts. Samples with elevated *S100A8* and *-A9* expression levels are represented by red lines. The log-rank test was used to calculate *p*-values. The shaded areas represent the 95% confidence interval.

d *S100A8* and *-A9* mRNA expression in primary breast tumor samples from the indicated cohorts, stratified by receptor status. The four datasets used include the three datasets described in Fig. 1 and Supplementary Fig. 1a, plus the TCGA Breast dataset. Each box representing a patient group has a number that indicates the sample size. Box plots show the 25th, 50th (median), and 75th percentiles. A pairwise two-tailed *t*-test between the respective groups was used to calculate *p*-values. NS: not significant.

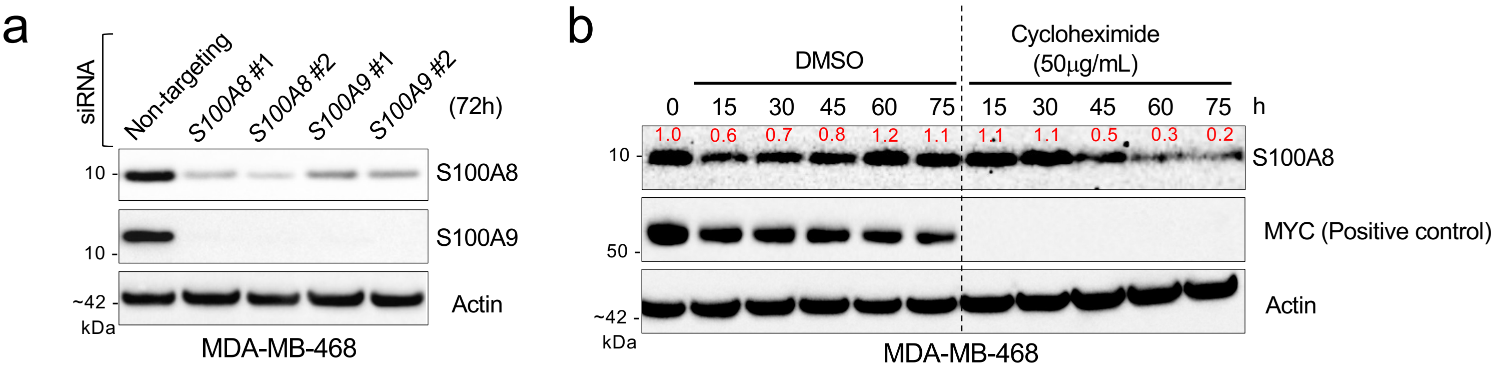

c

S100A8 vs.	TCGA Spearman's	TCGA p-value	METABRIC Spearman's	METABRIC p-value
FGFR1	-0.0426	0.223	-0.0141	0.539
FGFR2	-0.202	5.93E-09	-0.111	1.13E-06
FGFR3	-0.297	4.77E-18	-0.284	1.28E-36
FGFR4	0.201	6.74E-09	0.302	1.53E-41
PIM1	0.406	8.38E-34	0.372	1.56E-63
PIM2	0.3	2.15E-18	0.365	4.39E-61
PIM3	0.0877	0.0122	0.0449	0.0499
MTOR	-0.0895	0.0105	0	0.847
FLT1 (VEGFR1)	0.0846	0.0156	0.0105	0.646
KDR (VEGFR2)	0	0.892	-0.0129	0.574
SRC	0.181	1.88E-07	0.222	9.16E-23
ABL1	0.0163	6.41E-01	0.0489	0.0328
ABL2	0.0234	0.504	0.138	1.54E-09

S100A9 vs.	TCGA Spearman's	TCGA p-value	METABRIC Spearman's	METABRIC p-value
FGFR1	-0.255	0.466	0.0641	5.14E-03
FGFR2	-0.159	5.03E-06	-0.0834	2.69E-04
FGFR3	-0.317	1.59E-20	-0.0834	2.69E-04
FGFR4	0.204	3.72E-09	0.253	3.93E-29
PIM1	0.375	1.03E-28	0.391	1.04E-70
PIM2	0.268	6.64E-15	0.351	2.62E-56
PIM3	0.0744	0.0335	-0.0161	0.483
MTOR	-0.0943	7.02E-03	-0.0636	5.48E-03
FLT1 (VEGFR1)	0.046	0.189	0.0136	0.552
KDR (VEGFR2)	-0.0125	0.721	-0.0221	0.335
SRC	0.171	9.18E-07	0.164	5.15E-13
ABL1	0.0602	0.0855	-0.0246	0.284
ABL2	0.0209	0.55	0.135	3.65E-09

Supplementary Fig. 2. Protein stability of S100A8 and -A9 and correlations between their gene expression and that of *PIM kinases.*

a Representative western blots showing S100A8 and -A9 expression in the cells treated with the indicated siRNAs. Two siRNA sequences per gene were used as indicated. Actin serves as a loading control.

b Representative western blots showing the effects of cycloheximide on the protein abundance of S100A8 and MYC. MYC serves as a positive control for protein degradation. Actin serves as a loading control. The numbers in red indicate relative protein expression.

c Gene co-expression analysis of *S100A8* or -*A9* and the indicated genes in the TCGA breast (n = 817) and breast cancer METABRIC (n = 2,509) datasets.

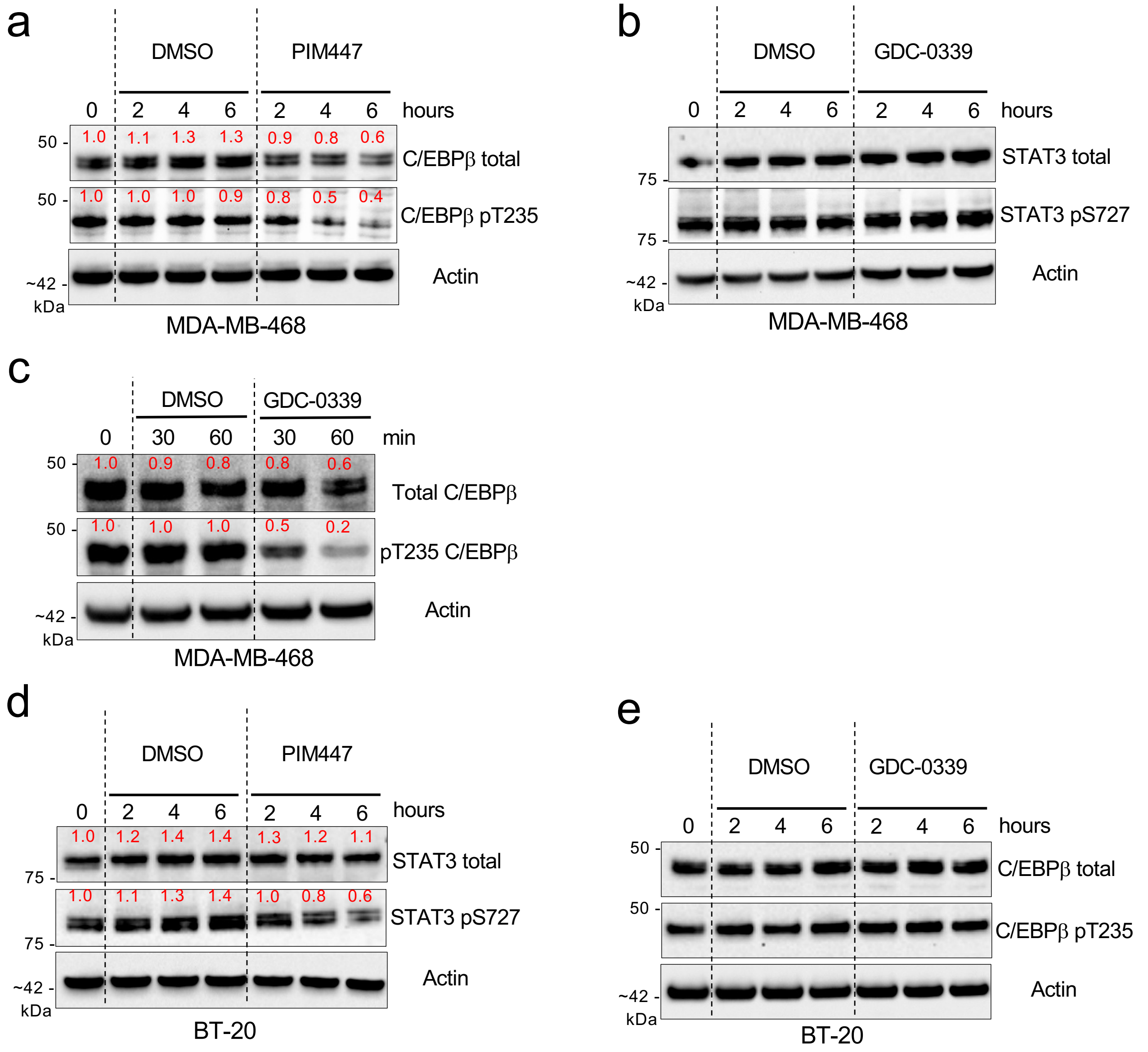

Supplementary Fig. 3. The effects of small molecule PIM kinase inhibition on total and phosphorylated C/EBPβ and STAT3 in human TNBC cell lines.

a Representative western blots showing the effects of PIM447 on the levels of total or T235-phosphorylated C/EBPβ in MDA-MB-468 cells.

b Representative western blots showing the effects of GDC-0339 on the levels of total or S727-phosphorylated STAT3 in MDA-MB-468 cells.

c Representative western blots showing the effects of GDC-0339 on the levels of total or T235-phosphorylated C/EBPβ in MDA-MB-468 cells.

d Representative western blots showing the effects of PIM447 on the levels of total or S727-phosphorylated STAT3 in BT-20 cells.

e Representative western blots showing the effects of GDC-0339 on the levels of total or T235-phosphorylated C/EBPβ in BT-20 cells.

Throughout this figure, PIM447 and GDC-0339 were used at 5 μM. Actin serves as a loading control. The numbers in red indicate relative protein expression.

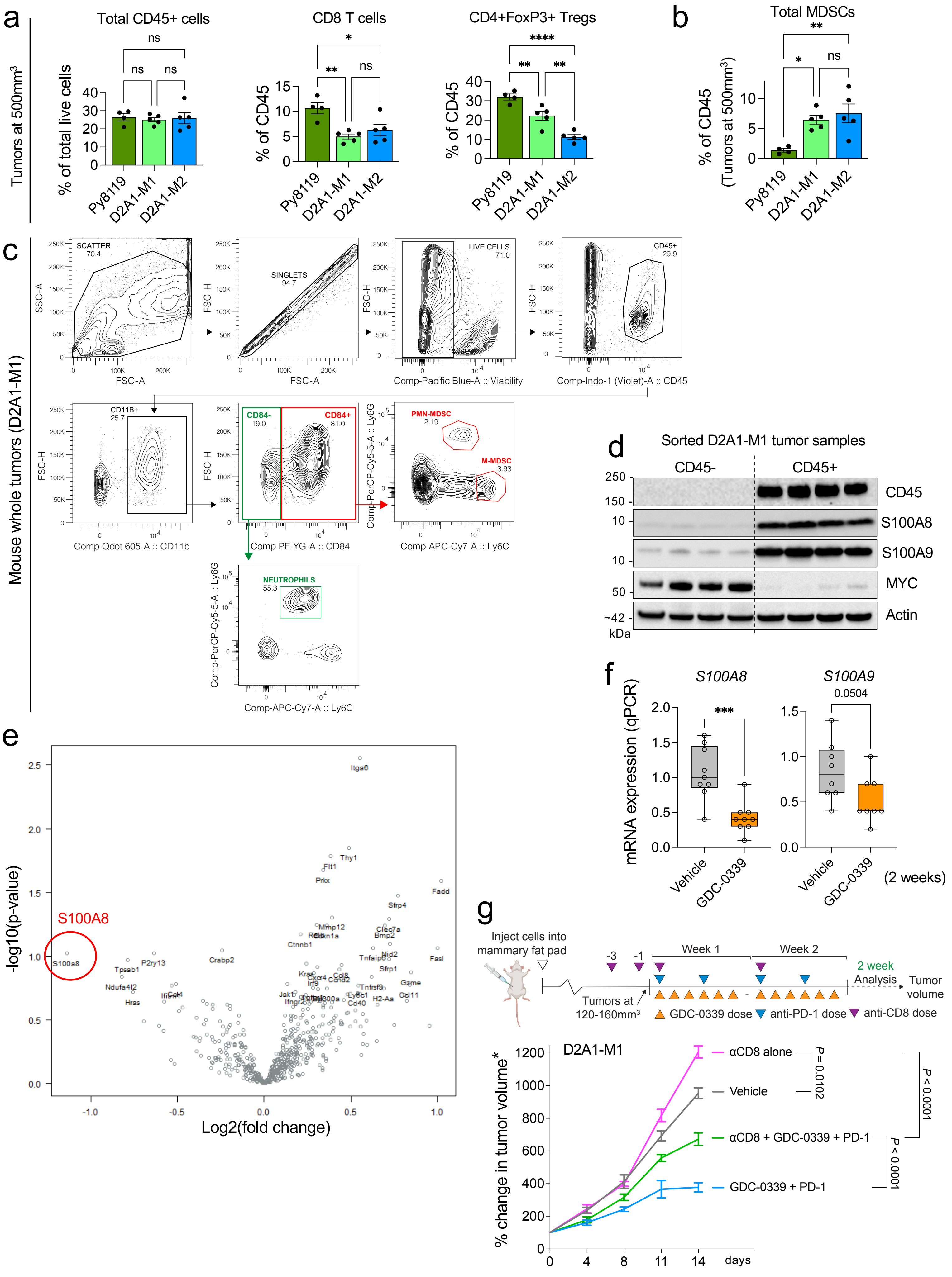

a
Tumors at 500mm³
Total CD45+ cells
% of total live cells
CD8 T cells
% of CD45
CD4+FoxP3+ Tregs
% of CD45
ns
*
**
****
Py8119
D2A1-M1
D2A1-M2
b
Total MDSCs
% of CD45 (Tumors at 500mm³)
c
Mouse whole tumors (D2A1-M1)
SCATTER 70.4
SINGLETS 94.7
LIVE CELLS 71.0
CD45+ 29.9
CD11B+ 25.7
CD84- 19.0
CD84+ 81.0
PMN-MDSC 2.19
M-MDSC 3.93
NEUTROPHILS 55.3
SSC-A
FSC-A
FSC-H
Comp-Pacific Blue-A :: Viability
Comp-Indo-1 (Violet)-A :: CD45
Comp-Qdot 605-A :: CD11b
Comp-PE-YG-A :: CD84
Comp-PerCP-Cy5-5-A :: Ly6G
Comp-APC-Cy7-A :: Ly6C
d
Sorted D2A1-M1 tumor samples
CD45-
CD45+
CD45
S100A8
S100A9
MYC
Actin
kDa
e
-log10(p-value)
Log2(fold change)
S100A8
Itga6
Thy1
Flt1
Prkx
Fadd
Sfrp4
Mmp12
Clec7a
Bmp2
Ctnnb1
Fasl
Crabp2
P2ry13
Tpsab1
Ndufa4l2
Hras
Ifi
Tnfaip
Sfrp1
Gzme
Ccl11
H2-Aa
Cd40
Ifngr2
Jak1
Irf9
Kras
Ccl8
Ccnd2
Tnfrsf9
f
mRNA expression (qPCR)
S100A8
S100A9
***
0.0504
Vehicle
GDC-0339
(2 weeks)
g
Inject cells into mammary fat pad
Week 1
Week 2
2 week Analysis
Tumor volume
Tumors at 120-160mm³
GDC-0339 dose
anti-PD-1 dose
anti-CD8 dose
D2A1-M1
% change in tumor volume*
days
αCD8 alone
Vehicle
αCD8 + GDC-0339 + PD-1
GDC-0339 + PD-1
P = 0.0102
P < 0.0001
P < 0.0001

Supplementary Fig. 4. Immunophenotyping of syngeneic orthotopic TNBC mouse tumors and the effects of PIM kinase inhibition on the expression of IO-related genes in vivo.

a, b Flow analysis showing the abundance of the indicated cell types in size-controlled tumors (n = 4 for Py8119, n = 5 for D2A1-M1 and –M2). MDSCs: myeloid-derived suppressor cells.

c Gating strategy for the identification of MDSCs in tumor samples. PMN-MDSCs: polymorphonuclear-MDSCs. M-MDSCs: monocytic-MDSCs.

d Representative western blots showing expression of the indicated proteins in the CD45- or CD45+ cell populations isolated from developed D2A1-M1 tumors (approximately 500 mm^3). Each lane represents two pooled CD45-sorted samples. MYC serves as a marker for TNBC cells. Actin serves as a loading control.

e Volcano plot showing the distribution of 770 IO-related genes, included in the NanoString IO360™ panel (mouse; NanoString Technologies, Inc.), in the indicated comparison group (n = 5 for the vehicle group, n = 3 for the GDC-0339 group).

f Relative mRNA levels of *S100A8* and -*A9* in the D2A1-M1 tumors treated with GDC-0339 or vehicle for 2 weeks (n = 9 per treatment group for *S100A8*, n = 8 per treatment group for *S100A9*). Box plots show the 25th, 50th (median), and 75th percentiles.

g Top: Schematic representation of the flow of the experiments. Bottom: Growth of the indicated tumors in mice treated with the indicated drugs or drug combinations (n = 6 per treatment group for the following groups: vehicle, anti-CD8 alone, and anti-CD8 + GDC-0339 + PD-1, and n = 5 for the GDC-0339 + PD-1 group). Linear regression analysis was used to calculate *p*-values. *Tumors at the time of initiating drug treatment, compared to those used in Fig 4e, were significantly larger (approx. 120-160 mm^3, as opposed to 75-100 mm^3).

Throughout this figure, error bars represent means +/- the standard error of the mean. Unless otherwise indicated, a two-tailed *t*-test was used to calculate *p*-values; *$p < 0.05$, **$p < 0.01$, ***$p < 0.001$, ****$p < 0.0001$. NS: not significant.

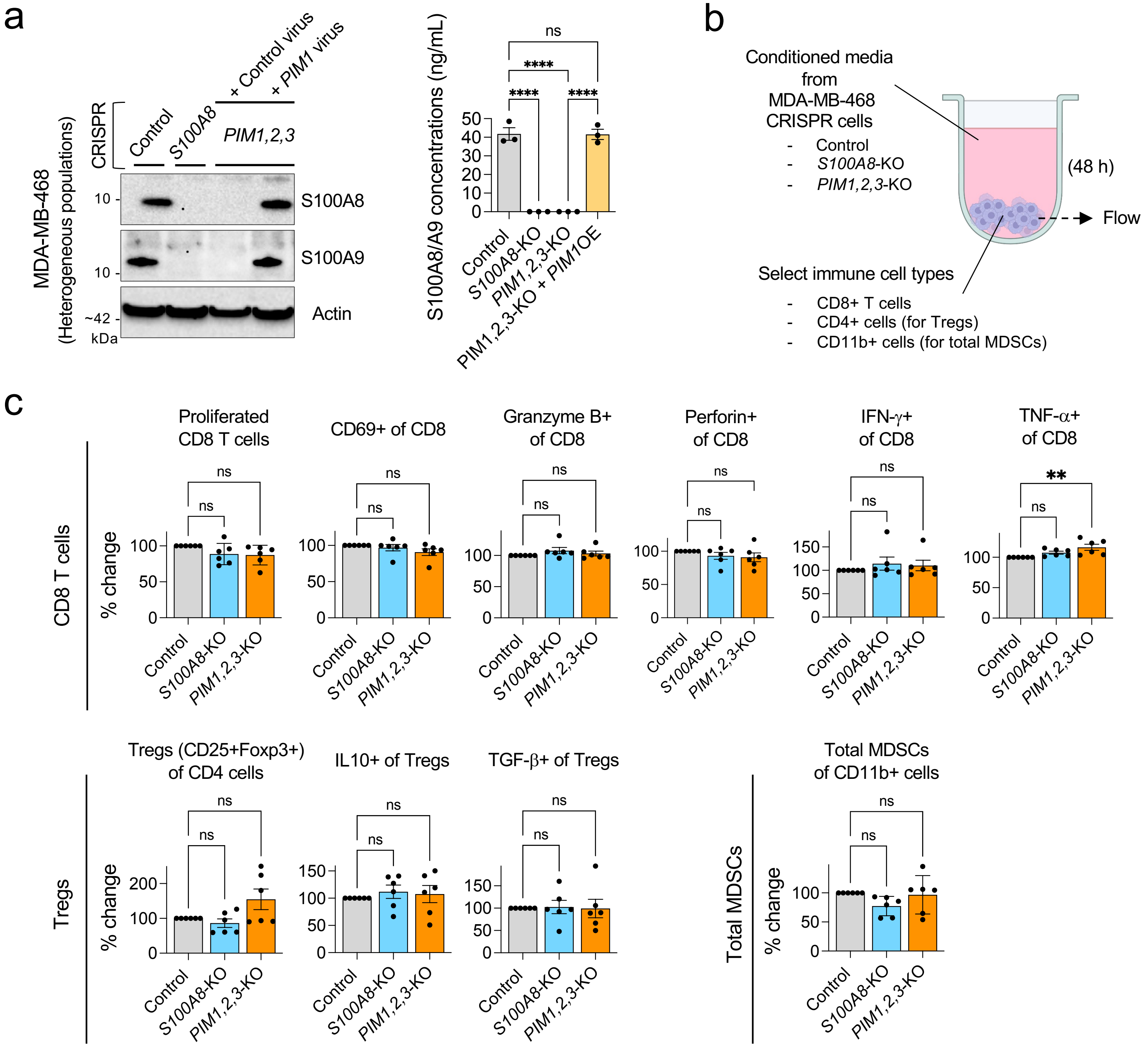

Supplementary Fig. 5. The effects of tumor expression of S100A8, -A9, and PIM kinases on the proliferation and activation of select human immune cell types in vitro.

a Left: Representative western blots showing expression of S100A8 and -A9 in the MDA-MB-468 cells that underwent the indicated genetic manipulations. Actin serves as a loading control. Right: S100A8/A9 secretion (48 h), as determined by ELISA, in the genetically modified MDA-MB-468 cells shown in (a) (n = 3). OE: overexpression.

b Schematic representation of the in vitro immune cell proliferation/activation assay.

c The effects of conditioned media from (a) on relative cell proliferation and activation levels of the indicated immune cell types (n = 6 per experimental group).

Throughout this figure, error bars represent means +/- the standard error of the mean. A two-tailed *t*-test was used to calculate *p*-values; $**p < 0.01$, $****p < 0.0001$. NS: not significant. KO: knockout. OE: overexpression. MDSCs: myeloid-derived suppressor cells.

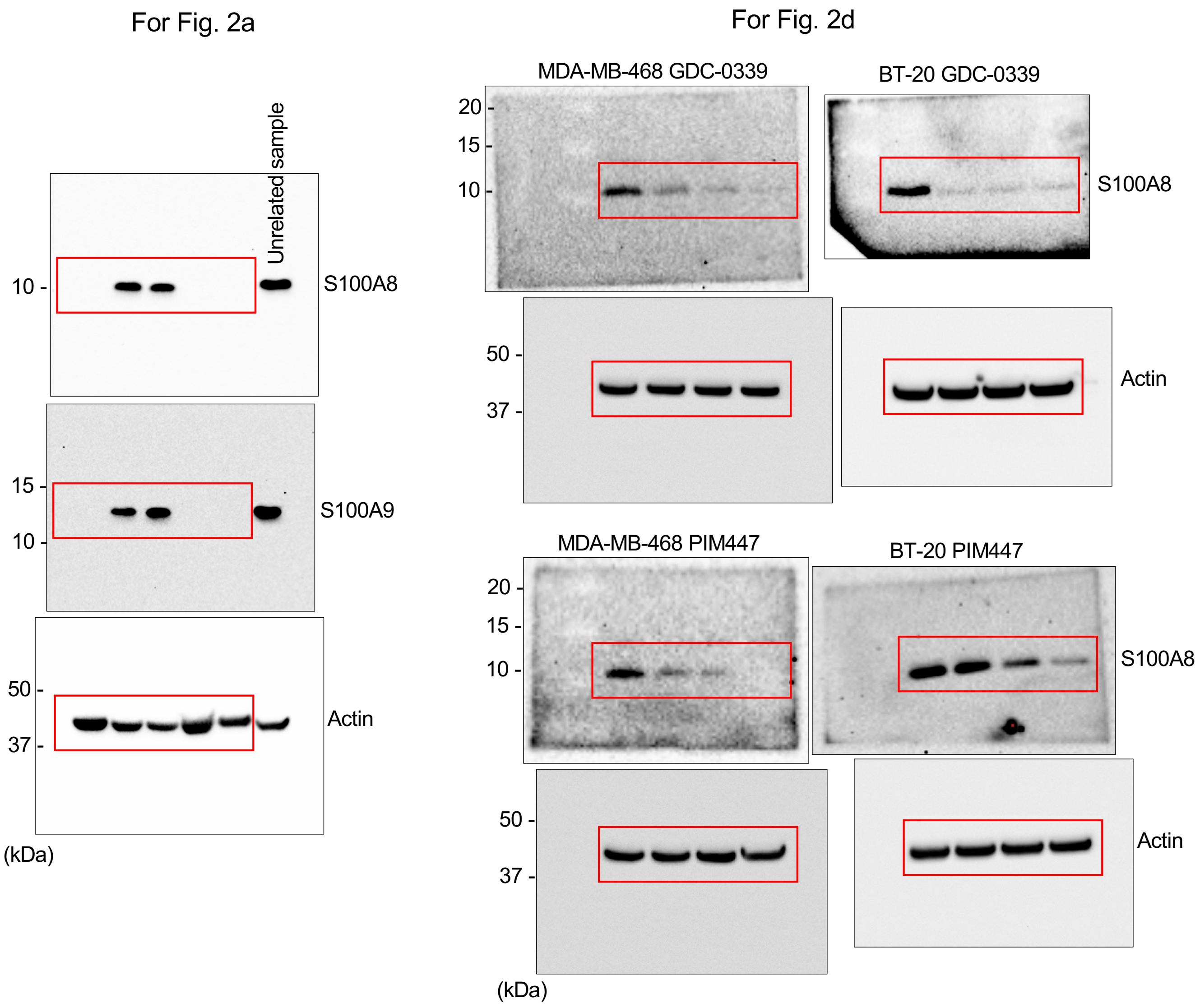

Supplementary Fig. 6 Unmodified digital images of chemiluminescence signals used to create the figure panels indicated. Western blot membranes were segmented to allow simultaneous detection of multiple targets with different molecular weights. The red rectangles show the areas used for the indicated figure panels. Molecular weights provided are estimates unless accompanied by a visible marker lane.

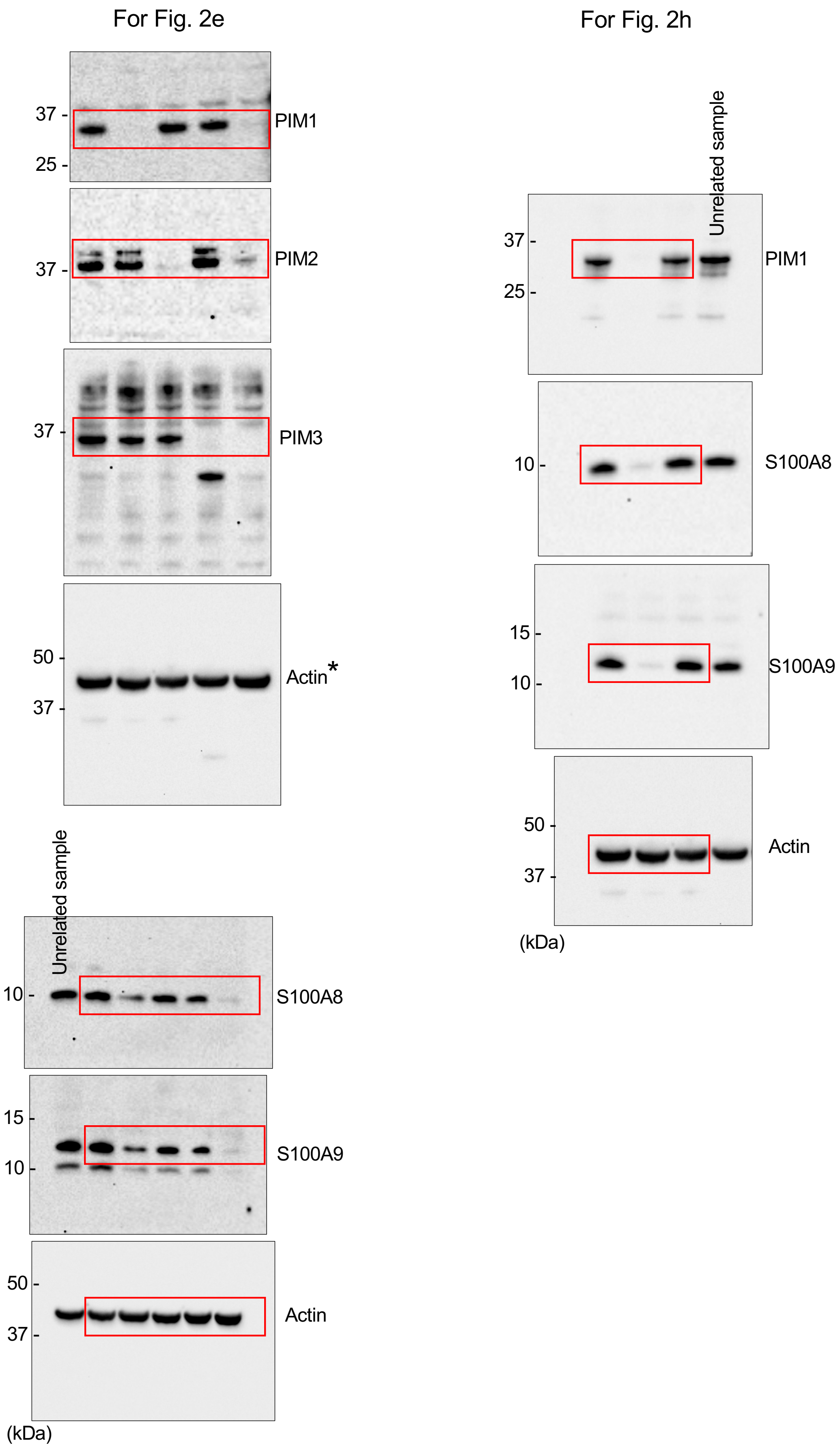

Supplementary Fig. 7 Unmodified digital images of chemiluminescence signals used to create the figure panels indicated. Western blot membranes were segmented to allow simultaneous detection of multiple targets with different molecular weights. The red rectangles show the areas used for the indicated figure panels. When different target proteins were probed on separate membranes, additional corresponding anti-Actin blots are provided. The blot indicated with an asterisk (*) was not used in the constructed figure panel and is provided for reference only. Molecular weights provided are estimates.

For Fig. 3c

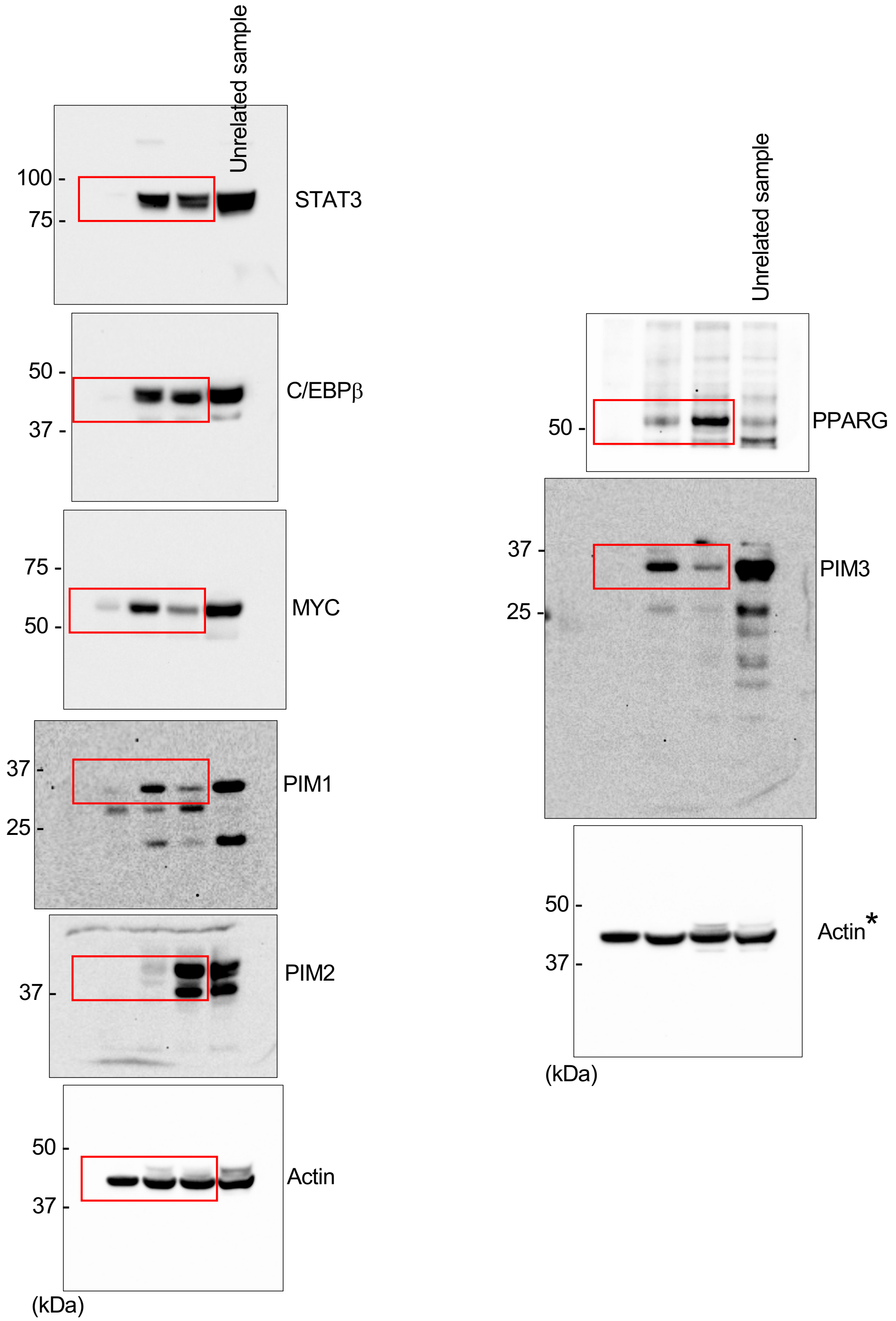

Supplementary Fig. 8 Unmodified digital images of chemiluminescence signals used to create the figure panels indicated. Western blot membranes were segmented to allow simultaneous detection of multiple targets with different molecular weights. The red rectangles show the areas used for the indicated figure panels. When different target proteins were probed on separate membranes, additional corresponding anti-Actin blots are provided. The blot indicated with an asterisk (*) was not used in the constructed figure panel and is provided for reference only. Molecular weights provided are estimates.

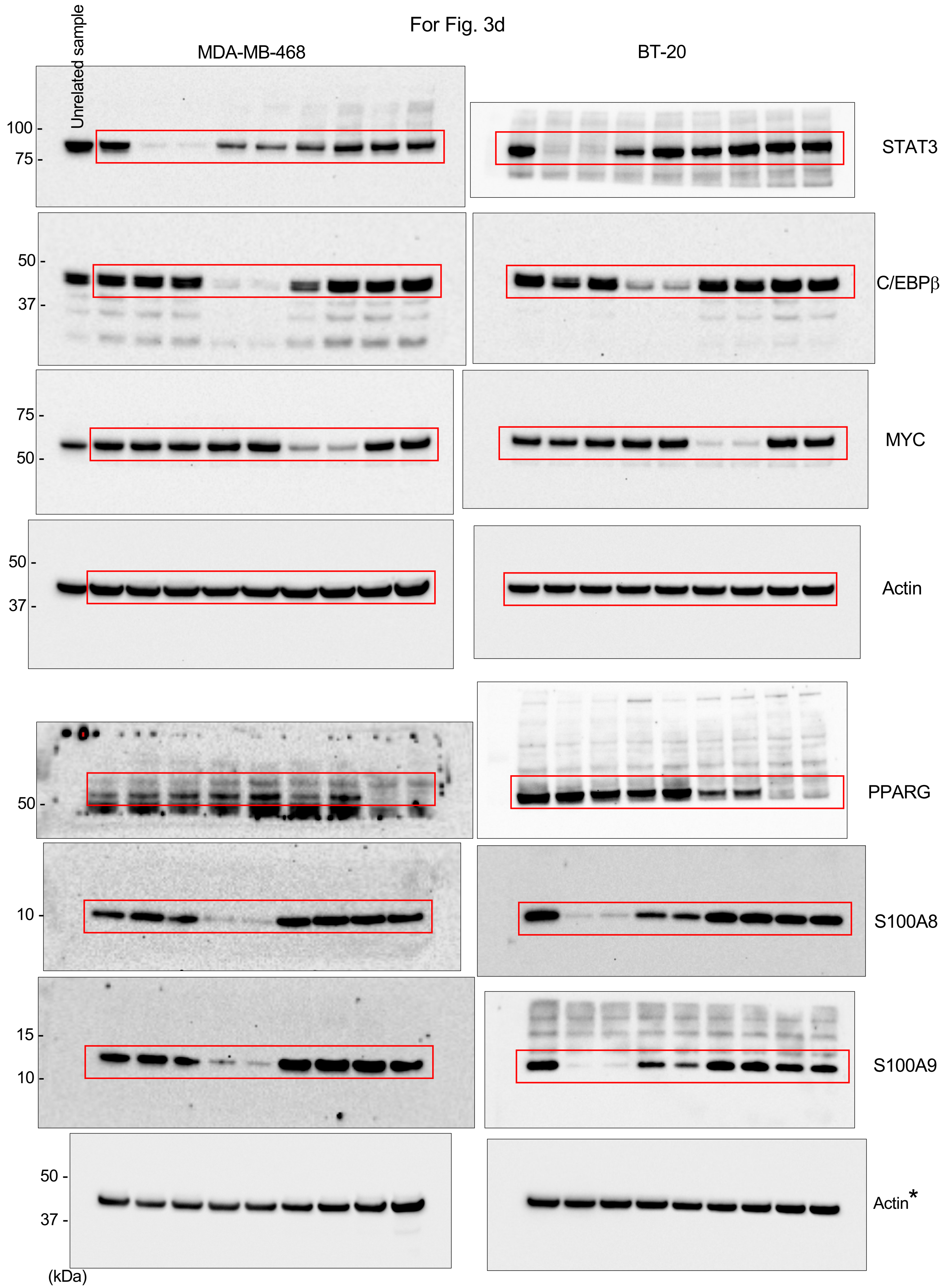

Supplementary Fig. 9 Unmodified digital images of chemiluminescence signals used to create the figure panels indicated. Western blot membranes were segmented to allow simultaneous detection of multiple targets with different molecular weights. The red rectangles show the areas used for the indicated figure panels. When different target proteins were probed on separate membranes, additional corresponding anti-Actin blots are provided. The blots indicated with an asterisk (*) were not used in the constructed figure panel and are provided for reference only. Molecular weights provided are estimates.

For Fig. 3e

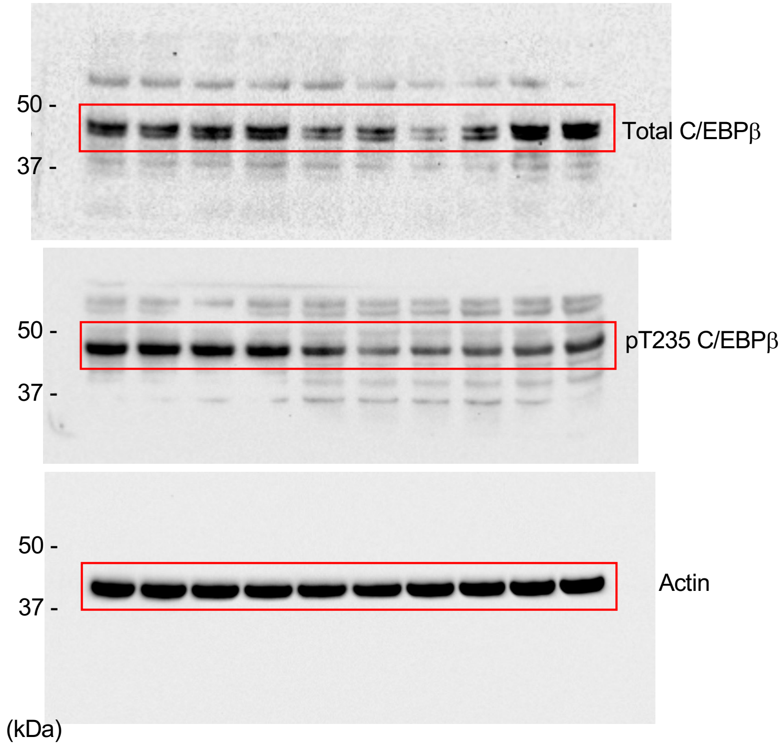

For Fig. 3f

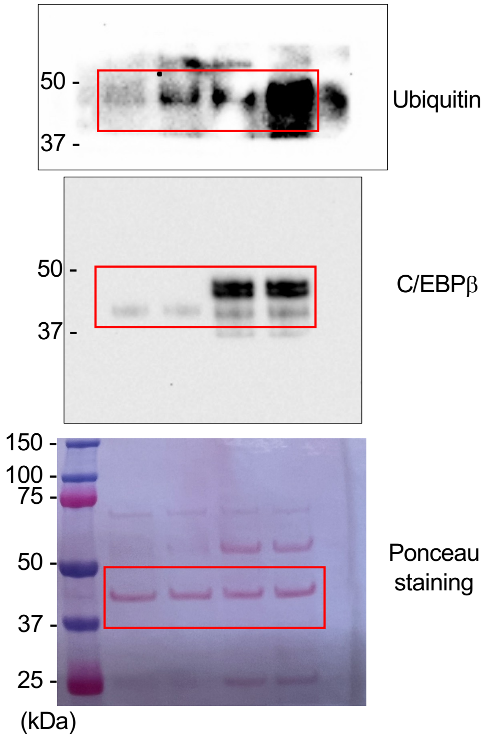

For Fig. 3g

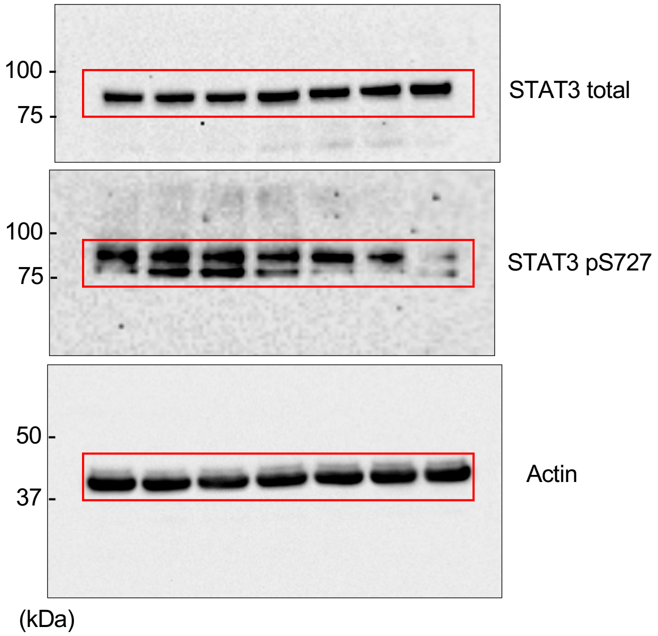

For Fig. 3h

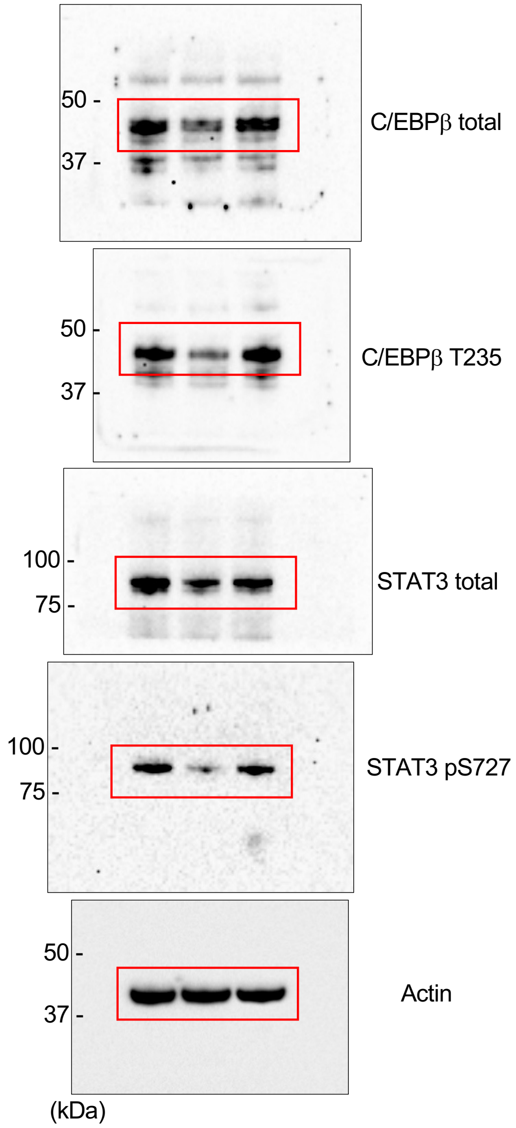

Supplementary Fig. 10 Unmodified digital images of chemiluminescence signals used to create the figure panels indicated. Western blot membranes were segmented to allow simultaneous detection of multiple targets with different molecular weights. The red rectangles show the areas used for the indicated figure panels. Molecular weights provided are estimates unless accompanied by a visible marker lane.

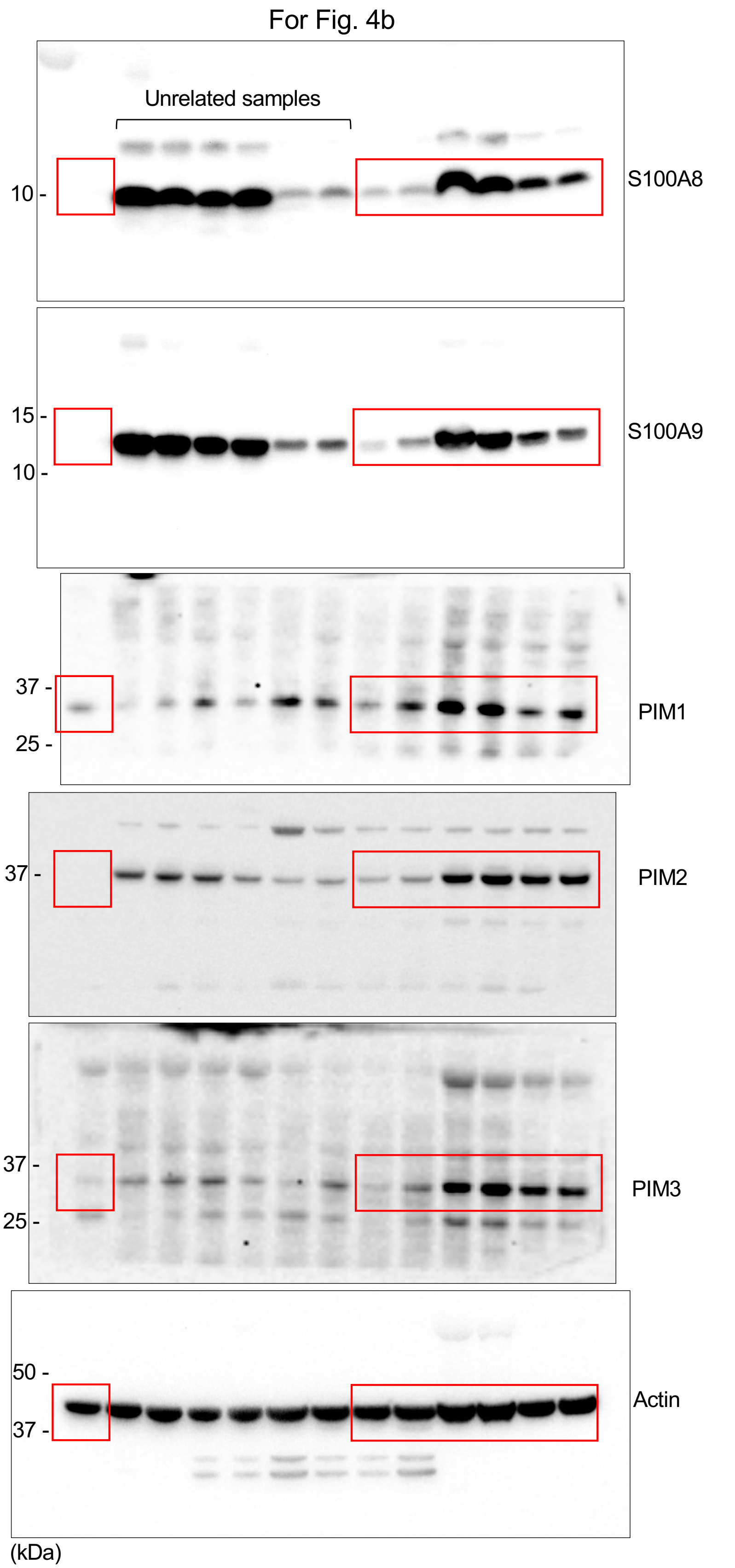

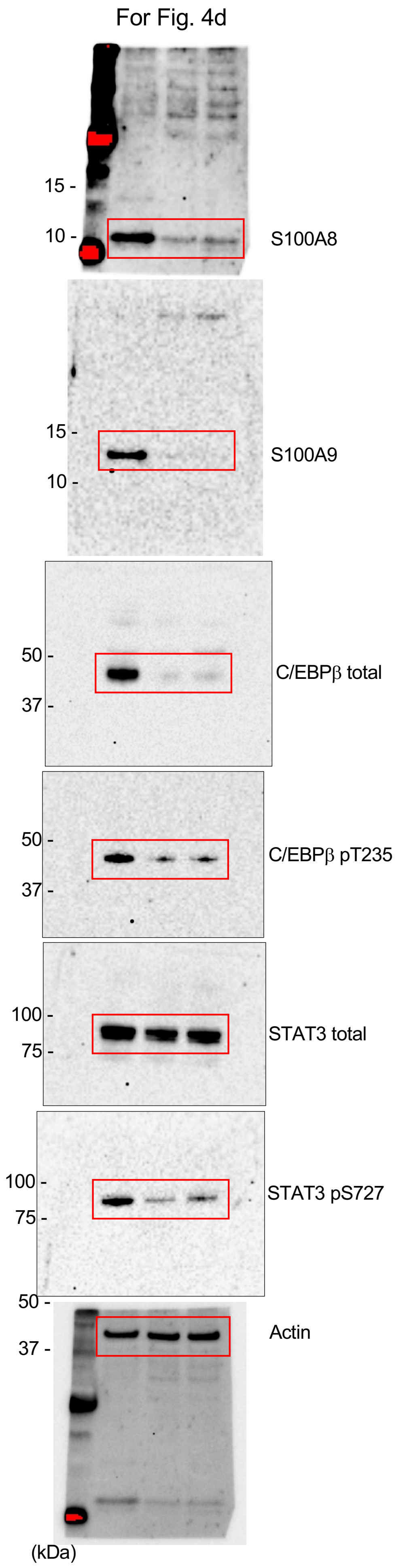

Supplementary Fig. 11 Unmodified digital images of chemiluminescence signals used to create the figure panels indicated. Western blot membranes were segmented to allow simultaneous detection of multiple targets with different molecular weights. The red squares and rectangles show the areas used for the indicated figure panels. Molecular weights provided are estimates unless accompanied by a visible marker lane.

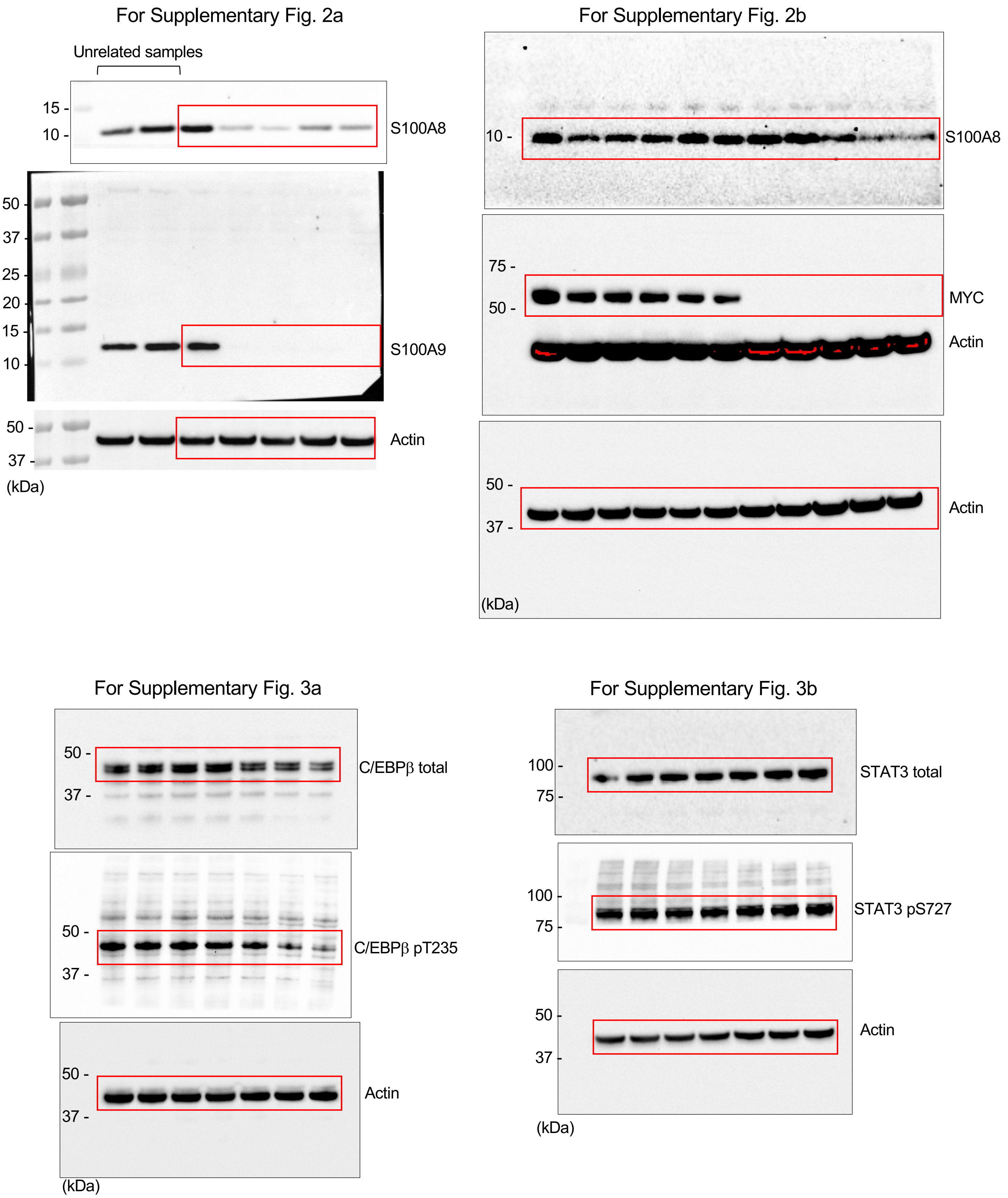

Supplementary Fig. 12 Unmodified digital images of chemiluminescence signals used to create the figure panels indicated. Western blot membranes were segmented to allow simultaneous detection of multiple targets with different molecular weights. The red rectangles show the areas used for the indicated figure panels. Molecular weights provided are estimates unless accompanied by a visible marker lane.

For Supplementary Fig. 3c

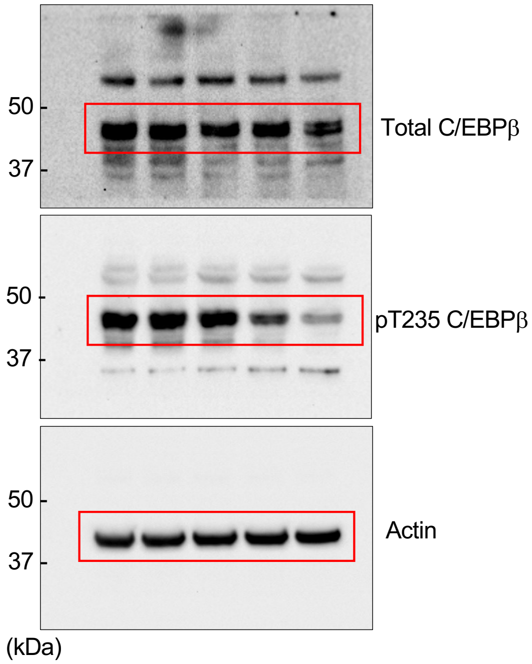

For Supplementary Fig. 3d

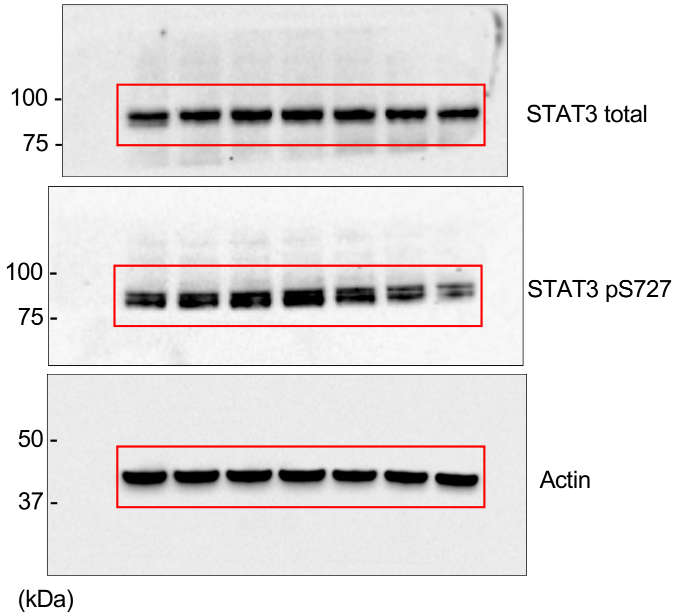

For Supplementary Fig. 3e

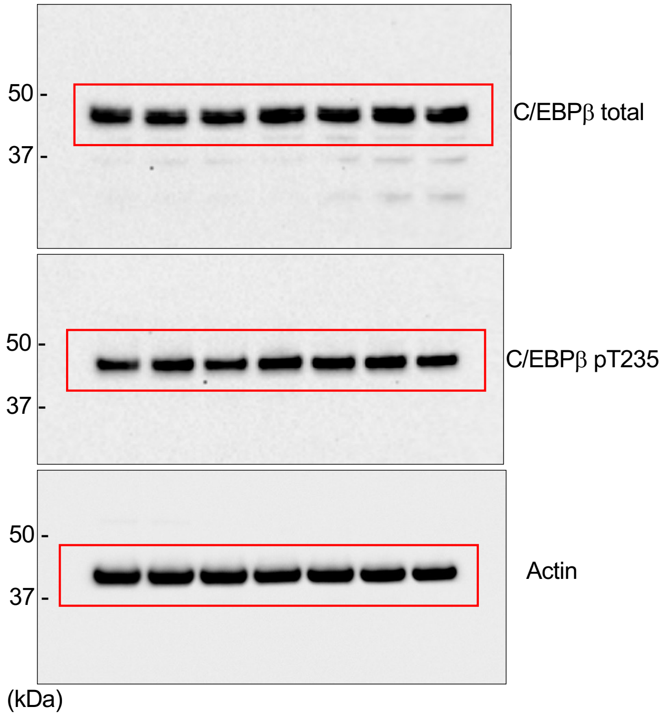

Supplementary Fig. 13 Unmodified digital images of chemiluminescence signals used to create the figure panels indicated. Western blot membranes were segmented to allow simultaneous detection of multiple targets with different molecular weights. The red rectangles show the areas used for the indicated figure panels. Molecular weights provided are estimates.

For Supplementary Fig. 4d

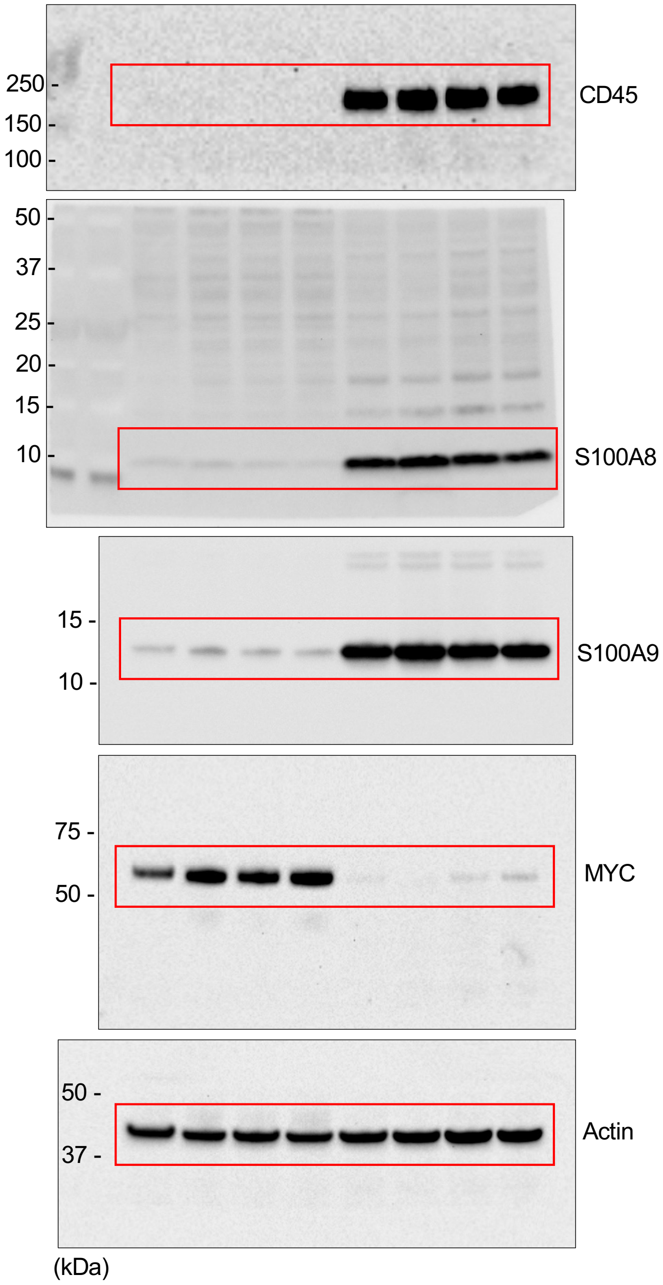

For Supplementary Fig. 5a

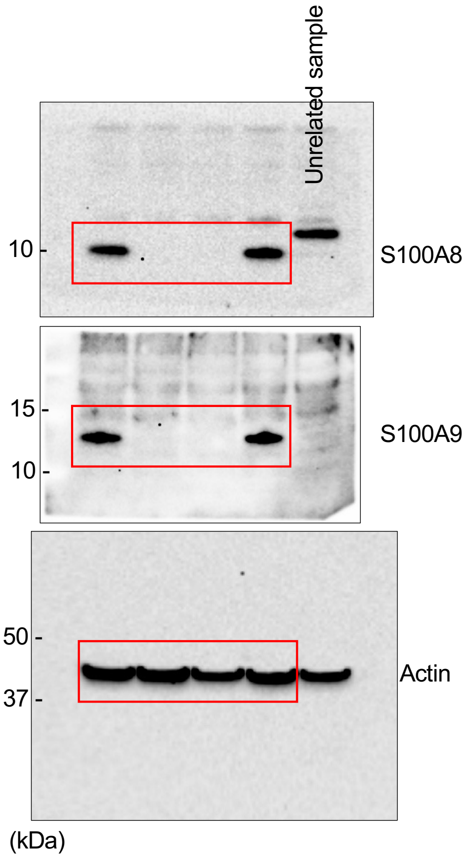

Supplementary Fig. 14 Unmodified digital images of chemiluminescence signals used to create the figure panels indicated. Western blot membranes were segmented to allow simultaneous detection of multiple targets with different molecular weights. The red rectangles show the areas used for the indicated figure panels. Molecular weights provided are estimates unless accompanied by a visible marker lane.

Compounds	Intended/known targets	Antiproliferative IC50 values (μM) in MDA-MB-468 cells (72 h)
GDC-0339	PIM1,2,3	>10
Erdafitinib	FGFR	2.8
PIM447	PIM1,2,3	8.02
Everolimus	mTOR- FKBP12	>10
Pazopanib	VEGFR 1/2/3; PDGFR-α/β	>10
Dasatinib	SRC; ABL	5
AMD1 (SAM486A)	AMD1	>10
CB-839	Glutaminase	>10
Dabrafenib	B-RafV600E; B-Raf	>10
Venetoclax	BCL2	>10
LDE225	Smo antagonist; Hedgehog	>10
Tazemetostat	EZH2	>10
Abemaciclib	CDK4/6	0.133
YM155	Survivin	0.0005
Capmatinib	cMET	>10
Alpelisib	PI3Kα/δ/γ	>10
Afuresertib	Akt1/2/3	>10
Bazedoxifene	ERα; ERβ	3.6
Prexasertib	CHK1/2; RSK	0.0003
GSK591	PRMT5	>10
I-BET-762	BET	>10
AZD6738	ATR	0.49
Cabozantinib	cMET; RET; KIT	4.6
Erlotinib	EGFR	7.5
Ganetespib	HSP90	0.0096
Crizotinib	ALK; MET	1.13
Olaparib	PARP1/2	7.75
Carfilzomib	20S proteasome	0.00052
Alisertib	Aurora A	0.167
Trametinib	MEK1/2	5.63
Panabinostat	HDAC	0.025

Supplementary Table 1. List of 30 targeted anticancer agents used in the initial screen, plus GDC-0339, their intended targets, and their antiproliferative half-maximal inhibitory concentration (IC50) values established in MDA-MB-468 cells by CellTiter-Glo assay.
