# Supplementary material for: S100A8/A9 predicts response to PIM kinase and PD-1/PD-L1 inhibition in triple-negative breast cancer mouse models
Source: Commun Med (Lond). 2024 Feb 20;4:22. doi: 10.1038/s43856-024-00444-8 (PMC10879183; doi:10.1038/s43856-024-00444-8)
Supplement: Supplementary file 3 — Description of Additional Supplementary Files [file 43856_2024_444_MOESM3_ESM.pdf]

# Description of Additional Supplementary Files

**File Name:** Supplementary Data 1
**Description:** Identified prognostic genes

**File Name:** Supplementary Data 2
**Description:** REACTOME pathway analysis

**File Name:** Supplementary Data 3
**Description:** Transcription factor prediction

**File Name:** Supplementary Data 4
**Description:** NanoString IO360TM raw counts

**File Name:** Supplementary Data 5
**Description:** Source data
