# Supplementary material for: S100A8/A9 predicts response to PIM kinase and PD-1/PD-L1 inhibition in triple-negative breast cancer mouse models
Source: Commun Med (Lond). 2024 Feb 20;4:22. doi: 10.1038/s43856-024-00444-8 (PMC10879183; doi:10.1038/s43856-024-00444-8)
Supplement: Supplementary file 9 — Reporting Summary [file 43856_2024_444_MOESM9_ESM.pdf]

Corresponding author(s): Dai Horiuchi

Last updated by author(s): 2023-11-22

Reporting Summary

Nature Portfolio wishes to improve the reproducibility of the work that we publish. This form provides structure for consistency and transparency in reporting. For further information on Nature Portfolio policies, see our Editorial Policies and the Editorial Policy Checklist.

Please do not complete any field with "not applicable" or n/a. Refer to the help text for what text to use if an item is not relevant to your study.
For final submission: please carefully check your responses for accuracy; you will not be able to make changes later.

Statistics

For all statistical analyses, confirm that the following items are present in the figure legend, table legend, main text, or Methods section.

n/a | Confirmed

- [Confirmed] The exact sample size (*n*) for each experimental group/condition, given as a discrete number and unit of measurement
- [Confirmed] A statement on whether measurements were taken from distinct samples or whether the same sample was measured repeatedly
- [Confirmed] The statistical test(s) used AND whether they are one- or two-sided
  Only common tests should be described solely by name; describe more complex techniques in the Methods section.
- [n/a] A description of all covariates tested
- [Confirmed] A description of any assumptions or corrections, such as tests of normality and adjustment for multiple comparisons
- [Confirmed] A full description of the statistical parameters including central tendency (e.g. means) or other basic estimates (e.g. regression coefficient) AND variation (e.g. standard deviation) or associated estimates of uncertainty (e.g. confidence intervals)
- [Confirmed] For null hypothesis testing, the test statistic (e.g. *F*, *t*, *r*) with confidence intervals, effect sizes, degrees of freedom and *P* value noted
  Give P values as exact values whenever suitable.
- [n/a] For Bayesian analysis, information on the choice of priors and Markov chain Monte Carlo settings
- [n/a] For hierarchical and complex designs, identification of the appropriate level for tests and full reporting of outcomes
- [Confirmed] Estimates of effect sizes (e.g. Cohen's *d*, Pearson's *r*), indicating how they were calculated

Our web collection on statistics for biologists contains articles on many of the points above.

Software and code

Policy information about availability of computer code

Data collection	BioTek Gen5 Software Program (Ver. 3.04) was used to acquire ELISA signals. BioRad Image Lab Touch Software (Ver. 2.3.0.07) was used to
Data analysis	R (Ver. 4.2.2) was used for all bioinformatics analyses. Prism (Ver. 9.5.0) was used for all statistical analyses. FlowJo (Ver. 10.6.2) was used to

For manuscripts utilizing custom algorithms or software that are central to the research but not yet described in published literature, software must be made available to editors and reviewers. We strongly encourage code deposition in a community repository (e.g. GitHub). See the Nature Portfolio guidelines for submitting code & software for further information.

Data

Policy information about availability of data

All manuscripts must include a data availability statement. This statement should provide the following information, where applicable:
- Accession codes, unique identifiers, or web links for publicly available datasets
- A description of any restrictions on data availability
- For clinical datasets or third party data, please ensure that the statement adheres to our policy

The four publicly available gene expression datasets used in this study are GSE25066, Yau (also identified in the literature as the chemotherapy-naïve historical dataset), Chin, and the TCGA breast invasive carcinoma datasets. The Yau, Chin, and TCGA breast datasets are available on the UCSC Cancer Browser or UCSC Xena (https://xenabrowser.net/datapages/). Additionally, the breast cancer METABRIC dataset is available in cBioPortal (http://www.cbioportal.org/datasets). The NanoString IO360 data file (raw counts) is included in the Supplementary Materials.

Human research participants

Policy information about studies involving human research participants and Sex and Gender in Research.

Reporting on sex and gender	N/A
Population characteristics	N/A
Recruitment	N/A
Ethics oversight	N/A

Note that full information on the approval of the study protocol must also be provided in the manuscript.

Field-specific reporting

Please select the one below that is the best fit for your research. If you are not sure, read the appropriate sections before making your selection.

(●) Life sciences ( ) Behavioural & social sciences ( ) Ecological, evolutionary & environmental sciences

Life sciences study design

All studies must disclose on these points even when the disclosure is negative.

Sample size	No statistical method was used to predetermine the sample size throughout this study. All cell-based in vitro experiments were performed in
Data exclusions	No data were excluded.
Replication	All cell-based in vitro experiments were independently repeated at least three times, as indicated in figure legends. Animal experiments were
Randomization	Tumor-bearing mice of similar tumor burden were equally divided into the control and experimental groups for subsequent drug treatment.
Blinding	Animal efficacy experiments, the NanoString gene expression analysis, and most in vitro experiments determining the study course were carried

Behavioural & social sciences study design

All studies must disclose on these points even when the disclosure is negative.

Study description	
Research sample	
Sampling strategy	
Data collection	
Timing	
Data exclusions	
Non-participation	
Randomization	

Ecological, evolutionary & environmental sciences study design

All studies must disclose on these points even when the disclosure is negative.

Study description	
Research sample	
Sampling strategy	
Data collection	
Timing and spatial scale	

Data exclusions

Reproducibility

Randomization

Blinding

Did the study involve field work? Yes No

Field work, collection and transport

Field conditions

Location

Access & import/export

Disturbance

Reporting for specific materials, systems and methods

We require information from authors about some types of materials, experimental systems and methods used in many studies. Here, indicate whether each material, system or method listed is relevant to your study. If you are not sure if a list item applies to your research, read the appropriate section before selecting a response.

Materials & experimental systems

n/a Involved in the study

- Antibodies
- Eukaryotic cell lines
- Palaeontology and archaeology
- Animals and other organisms
- Clinical data
- Dual use research of concern

Methods

n/a Involved in the study

- ChIP-seq
- Flow cytometry
- MRI-based neuroimaging

Antibodies

Antibodies used: Western analysis:

Validation: Most antibodies used for western blot analysis were validated through a combination of CRISPR and siRNA experiments, as depicted in

Eukaryotic cell lines

Policy information about cell lines and Sex and Gender in Research

Cell line source(s): All human TNBC cell lines used in this study were directly purchased from ATCC and were used within approximately 30-40

Authentication: All cell lines were from early passages of an authenticated ATCC stock and were not authenticated during this study.

Mycoplasma contamination: The Universal Mycoplasma Detection Kit (ATCC, 30-1012K) was used to ensure that cells were not infected with mycoplasma.

Commonly misidentified lines (See ICLAC register): This study did not use commonly misidentified lines.

Palaeontology and Archaeology

Specimen provenance

Specimen deposition

Dating methods

☐ Tick this box to confirm that the raw and calibrated dates are available in the paper or in Supplementary Information.

Ethics oversight

Note that full information on the approval of the study protocol must also be provided in the manuscript.

Animals and other research organisms

Policy information about studies involving animals; ARRIVE guidelines recommended for reporting animal research, and Sex and Gender in Research

Laboratory animals	BALB/cJ and C57BL/6J mice, aged 10-12 weeks
Wild animals	This study did not involve wild animals.
Reporting on sex	This study focused primarily on triple-negative breast cancer (TNBC) and thus utilized exclusively female mice.
Field-collected samples	This study did not involve samples collected from the field.
Ethics oversight	Northwestern University Institutional Animal Care and Use Committee

Note that full information on the approval of the study protocol must also be provided in the manuscript.

Clinical data

Policy information about clinical studies

All manuscripts should comply with the ICMJE guidelines for publication of clinical research and a completed CONSORT checklist must be included with all submissions.

Clinical trial registration	
Study protocol	
Data collection	
Outcomes	

Dual use research of concern

Policy information about dual use research of concern

Hazards

Could the accidental, deliberate or reckless misuse of agents or technologies generated in the work, or the application of information presented in the manuscript, pose a threat to:

No | Yes
- Public health
- National security
- Crops and/or livestock
- Ecosystems
- Any other significant area

Experiments of concern

Does the work involve any of these experiments of concern:

No | Yes
- Demonstrate how to render a vaccine ineffective
- Confer resistance to therapeutically useful antibiotics or antiviral agents
- Enhance the virulence of a pathogen or render a nonpathogen virulent
- Increase transmissibility of a pathogen
- Alter the host range of a pathogen
- Enable evasion of diagnostic/detection modalities
- Enable the weaponization of a biological agent or toxin
- Any other potentially harmful combination of experiments and agents

ChIP-seq

Data deposition

- [ ] Confirm that both raw and final processed data have been deposited in a public database such as GEO.
- [ ] Confirm that you have deposited or provided access to graph files (e.g. BED files) for the called peaks.

Data access links
May remain private before publication.

Files in database submission

Genome browser session (e.g. UCSC)

Methodology

Replicates

Sequencing depth

Antibodies

Peak calling parameters

Data quality

Software

Flow Cytometry

Plots

Confirm that:

- [x] The axis labels state the marker and fluorochrome used (e.g. CD4-FITC).
- [x] The axis scales are clearly visible. Include numbers along axes only for bottom left plot of group (a 'group' is an analysis of identical markers).
- [x] All plots are contour plots with outliers or pseudocolor plots.
- [x] A numerical value for number of cells or percentage (with statistics) is provided.

Methodology

Sample preparation: For mouse samples, tumors were minced and dissociated using the Mouse Tumor Dissociation Kit (Miltenyi

Instrument: Data were acquired on LSRFortessa Cell Analyzer (BD Biosciences) equipped with 355nm, 405nm, 488nm, 561nm, and 640nm

Software: FACSDiva (BD Biosciences, version 8.0.1) was used for data acquisition. Data were analyzed using FlowJo (version 10.6.2).

Cell population abundance: At least 50,000 total events per sample were analyzed.

Gating strategy: All experiments/panels were compensated using single-color controls.

- [x] Tick this box to confirm that a figure exemplifying the gating strategy is provided in the Supplementary Information.

Magnetic resonance imaging

Experimental design

Design type

Design specifications

Behavioral performance measures

Acquisition

Imaging type(s)

Field strength

Sequence & imaging parameters

Area of acquisition

Diffusion MRI ○ Used ○ Not used

Preprocessing

Preprocessing software

Normalization

Normalization template

Noise and artifact removal

Volume censoring

Statistical modeling & inference

Model type and settings

Effect(s) tested

Specify type of analysis:

Whole brain ROI-based Both

Statistic type for inference
(See Eklund et al. 2016)

Correction

Models & analysis

n/a	Involved in the study
	Functional and/or effective connectivity
	Graph analysis
	Multivariate modeling or predictive analysis

Functional and/or effective connectivity

Graph analysis

Multivariate modeling and predictive analysis

This checklist template is licensed under a Creative Commons Attribution 4.0 International License, which permits use, sharing, adaptation, distribution and reproduction in any medium or format, as long as you give appropriate credit to the original author(s) and the source, provide a link to the Creative Commons license, and indicate if changes were made. The images or other third party material in this article are included in the article's Creative Commons license, unless indicated otherwise in a credit line to the material. If material is not included in the article's Creative Commons license and your intended use is not permitted by statutory regulation or exceeds the permitted use, you will need to obtain permission directly from the copyright holder. To view a copy of this license, visit http://creativecommons.org/licenses/by/4.0/
